# 

**Published:** 1903-09

**Authors:** 


*The following donations have been received since the publication of the List in June :*

*August 31st, 1903.*

C. Elliott, M.D. (1) ... ... ... ... ... ... ... 37 volumes.
James Fawn and Son (2) ... ... ... ... ... ... 7 ,,
R. Shingleton Smith, M.D. (3) ... ... ... ... ... 1 volume.
Rhode Island Medical Society (4) ... ... ... ... 1 ,,

### FORTY-NINTH LIST OF BOOKS.

The titles of books mentioned in previous lists are not repeated.

The figures in brackets refer to the figures after the names of the donors, and show by whom the volumes were presented. The books to which no such figures are attached have either been bought from the Library Fund or received through the *Journal.*

**Albinus, B. S.** ... *Explicatio Tabularum anatomicarum B. Eustachii* (1) 1744
**Andrewes, F. W.** *Lessons in Disinfection and Sterilisation* ... ... ... 1903
**Armstrong and J. E. Harburn, W.** *Buxton* ... ... ... ... ... ... ... 1903
**Beaney, J. G.** ... *Contributions to Conservative Surgery* ... ... ... (2) 1859
**Bell, J.** ... ... *The Principles of Surgery* (2). New Ed. by C. Bell. 4 vols. 1826
**Burdett, Sir H.** ... *The Medical Attendance of Londoners* ... ... ... ... 1903
**Caldwell, W. A. Pusey and E. W.** *The Röntgen Rays in Therapeutics and Diagnosis* ... ... ... ... ... ... ... ... 1903
**Carter and A. H. Cheatle, R. B.** *Sight and Hearing in Childhood* ... ... [N.D.]
**Cheatle, R. B. Carter and A. H.** *Sight and Hearing in Childhood* ... ... [N.D.]
**Dowse, T. S.** ... *Lectures on Massage and Electricity* ... ... 4th Ed. 1903
*Encyclopædia Medica.* (Ed. by C. Watson). Vols. XII., XIII. ... ... 1903
**Harburn, W. Armstrong and J. E.** *Buxton* ... ... ... ... ... ... ... 1903
**Harty, W.** ... ... *An Historic Sketch of the Contagious Fever in Ireland,* 1817-19 ... ... ... ... ... ... ... ... (1) 1820
**Herschell, G.** ... *Manual of Intragastric Technique* ... ... ... ... ... 1903
,, ... *Polyphase Currents in Electrotherapy* ... ... ... ... 1903
**Hewlett, R. T.** ... *Serum Therapy* ... ... ... ... ... ... ... ... ... 1903
**Higgins, C.** ... ... *Ophthalmic Practice.* (Ed. by A. W. Ormond.) 2nd Ed. 1903
**Hooper, R.** ... ... *Lexicon Medicum ; or, Medical Dictionary.* (1) 8th Ed. 1848
**Jahr [G. H. G.]** ... *New Manual of Homœopathic Medicine.* (2) 3rd American Ed. 2 vols. ... ... ... ... 1850-51
**Maddock, A. B.** ... *Pulmonary Consumption* ... ... ... ... (1) 5th Ed. 1854
**Magennis, E.** ... *Eye Symptoms as Aids in Diagnosis* ... ... ... ... 1903
**Monro, T. K.** ... *Manual of Medicine* ... ... ... ... ... ... ... 1903

| | | | |
|---|---|---|---|
| **Palmer, Margaret D.** | *Massage* | 2nd Ed. | 1903 |
| **Parsons, J. H.** | *The Ocular Circulation* | | 1903 |
| **Pusey and E. W. Caldwell, W. A.** | *The Röntgen Rays in Therapeutics and Diagnosis* | | 1903 |
| **Sinclair, W. J.** | *The Midwives Act*, 1902 | | 1903 |
| **Startin, J.** [Ed.] | *A Pharmacopœia for Diseases of the Skin* | 5th Ed. | 1903 |
| **Stevens, A. A.** | *Modern Materia Medica and Therapeutics* | 3rd Ed. | 1903 |
| **Sutton, J. B.** | *Tumours, Innocent and Malignant* | 3rd Ed. | 1903 |

### TRANSACTIONS, REPORTS, JOURNALS, &c.

| | | |
|---|---|---|
| American Journal of the Medical Sciences, The | (3) Vol. CXXIV. | 1902 |
| American Practitioner and News | Vols. XXXIII., XXXIV. | 1902 |
| Annali di Ostetricia e Ginecologia | | 1902 |
| Archivio di Ortopedia | | 1902 |
| Birmingham Medical Review, The | Vols. LI., LII. | 1902 |
| Bristol Health Report for 1902 | | 1903 |
| College of Physicians of Philadelphia, Transactions of the | Vol. XXIV. | 1902 |
| Edinburgh Medical Journal, The | N.S., Vol. XIII. | 1903 |
| Hospital, The | Vol. XXXIII. | 1903 |
| Hospitals— | | |
| St. Bartholomew's Hospital—Statistical Tables, 1902 | | 1903 |
| St. Thomas's Hospital Reports | Vol. XXX. | 1903 |
| Journal of the Sanitary Institute | Vol. XXIII. | 1902 |
| Laryngoscope, The | Vol. XII. | 1902 |
| Library, The | Vol. III. | 1902 |
| Library Association Record, The | Vols. III., IV. | 1901-02 |
| Louisville Monthly Journal, The | Vol. IX. | 1902-03 |
| Medical Chronicle, The | 4th Ser., Vol. IV. | 1902-03 |
| Nordischen Kongresses für innere Medizin, Verhandlungen des Vierten, 1902 | | 1903 |
| Northumberland and Durham Medical Journal, The | | 1902 |
| Obstetrical Transactions | Vol. XLIV. | 1902 |
| Philadelphia County Medical Society, Proceedings of the | Vol. XXIII. | 1902 |
| Practitioner, The | Vol. LXIX. | 1902 |
| Rhode Island Medical Society, Transactions of the | (4) Vol. VI., Part 4 | 1903 |
| Treatment | Vol. VI. | 1903 |
| University of Penna. Medical Bulletin | Vol. XV. | 1903 |

### Local Medical Notes.

**University College, Bristol.**—In the May examination for the F.R.C.S. Eng. diploma Messrs. R. F. Moorshead, M.B., B.S., and H. S. Jenkins, M.B., were successful. The following gentlemen have passed the primary F.R.C.S. examination: Messrs. W. J. H. Pinniger, M. H. Phillips, and C. F. Walters.

Mr. A. Coleridge has been appointed Assistant Resident Medical Officer to the North London Hospital for Consumption, Hampstead.

**University of London.**—At the M.B. Intermediate Examination the following have passed in the second division:—Messrs. J. F. Blackett, R. E. Thomas, and A. J. M. Wright.

**Bristol General Hospital.**—Mr. H. G. Kyle, M.B., B.Ch. Oxon., has been appointed Surgeon to the Hospital, and Mr. E. Hey Groves, M.D., B.Sc. Lond., Assistant-Surgeon.

**Long Fox Memorial Fund.**—We are glad to find that the work of the Honorary Secretaries to this Fund has been successful. A sum of £680 12s. 10d. has been collected. In accordance with the decision of the subscribers, a Memorial Tablet has been placed in the Cathedral, and arrangements have been made, in conjunction with the Board of Education, to found an Annual Lecture at University College, Bristol, on some subject connected with Medical Science, to be called the "Long Fox Lecture," and to establish a fund to be devoted to the assistance of a Medical Missionary Student.

**Bristol Royal Hospital for Sick Children and Women.**—The sudden death of Mr. Mark Whitwill on August 6th, 1903, removes from our midst one who has done much for the welfare of his city, and more particularly of the sick children and women who have derived benefit from the Hospital of which he was the founder more than forty years ago, and over which he has exercised a daily guardianship ever since. The never-ceasing interest and work of Mr. Whitwill in maintaining and improving the efficiency of this Hospital is to be commemorated by a proposed endowment of an existing ward at a cost of four thousand pounds. Such an enterprise would be thoroughly in accord with his own wishes, and we have no doubt that the sum required will be duly provided.

**Royal Medical Benevolent College.**—Several local medical men attended the Festival Dinner held on June 10th, when His Royal Highness the Prince of Wales presided over a goodly company. He mentioned that the Council of the College were anxious to raise the sum of £10,000 in order to place the finances of the Institution on a sound basis, and stated that a sum of over £6,500 had been collected. Mr. Paul Bush, our local honorary secretary, informs us that the list of contributions is being kept open in the hope that the sum required may be eventually realised. The 6th of July was the fiftieth anniversary of the laying of the foundation stone of the College, and the present is a fitting opportunity for making a strenuous effort to free the Institution from financial embarrassment. The fifty pensioners receive annuities of £30, and the fifty scholars are educated, boarded and clothed free of charge. About £8,000 a year is required to maintain the benevolent side of the Institution, and as there is not much in the way of assured income, fresh subscribers are urgently required if the good work is to be carried out as fully in the future as it has been in the past.

